# Radiography: A Plea for the Adoption of a Standard System of Units

**Published:** 1912-11-30

**Authors:** Alfred C. Norman

**Affiliations:** House Surgeon at Durham County Eye Infirmary.


### ELECTRICITY IN MODERN MEDICINE.*

### XXII.—Radiography: A Plea for the Adoption of a Standard System of Units.

By ALFRED C. NORMAN, M.D. Edin., House Surgeon at Durham County Eye Infirmary.

Radiography consists in making a permanent photographic record of the shadow images cast by the *x*-rays in passing through substances of different density. Instead of producing fluorescence the rays exert an invisible chemical action on the plate which can subsequently be converted into a visible image by a process known as development.

Radiography is nowadays quite as much an exact science as, say, chemistry. All the factors which go to the making of a successful radiograph can be expressed in standard units, they can all be directly measured and they are all under the control of the operator. If I make a good lateral radiograph of the orbit and accessory sinuses of a male adult by giving an exposure of thirty *seconds*, using a current of two *milliamperes* through a tube of No. 8 *penetration* (Wehnelt), with the anticathode 18 *inches* from the plate, I know that I can always obtain a similar result by giving the same exposure under similar conditions. And yet how often, in this country at any rate, we see radiography carried out purely by rule-of-thumb! In very few *x*-ray departments are the radiometer and the milliamperemeter both systematically used, while in some even a watch is dispensed with. Is there any wonder, then, that the radiographer in charge obtains one good result in about ten exposures, and that he takes each new radiograph with the pious hope, instead of with the absolute certainty, "that it will be a good one"?

I have discussed at considerable length in previous sections the methods of measuring and controlling the various radiographic units, and if beginners who read these articles are induced thereby to adopt exact and scientific methods, I shall not have written in vain, for their radiographic work will become a fascinating study instead of being a constant source of worry and doubt. One sees far too often in English and American text-books (even in some of the very latest) loose particulars of the radiographs illustrating the context, which are absolutely useless either as a basis of comparison or as a guide to beginners who wish to obtain similar results. It is useless, for instance, to state that a certain result was obtained from an exposure of twenty seconds, using five amperes through the *primary* of the coil, for with different apparatus and with tubes of different resistance this might mean that a current of two or one of ten milliamperes was passing through the *x*-ray tube.

### The Method of Taking a Radiograph.

In taking a radiograph no camera or lens is required, for the *x*-rays cannot be refracted by any known substance. The photograph plate, enclosed in paper envelopes, which are opaque to ordinary light but transparent to the *x*-rays, is placed beneath the part of the body to be radiographed and the *x*-ray tube is placed above it, so that the centre of the anticathode is directly over the centre of the part to be radiographed. A suitable current is passed through the tube for a certain length of time, the plate is then taken to the dark room, removed from its envelopes, developed and fixed.

The actual manipulations, then, are exceedingly simple, but the estimation of the correct exposure and the selection of a tube of suitable penetration will at first require a good deal of thought on the part of the worker. The factors that influence the exposure are: the current passing through the tube, the distance between anticathode and plate, the thickness of the part to be radiographed, and the penetrating power of the rays. We will now separately consider each of these factors with a view to reducing the problem of exposure to its simplest proportions.

### Current Passing Through the Tube.

We have already seen that the number of milliamperes (as shown by a d'Arsonval meter) passing through the *x*-ray tube is an exact indication of the quantity of *x*-rays that are being emitted by that tube at a given moment and that the *total* quantity of rays emitted further depends upon the *time* the current is allowed to run. Now, if we multiply the time in seconds by the number of milliamperes we arrive at a unit which has a constant and definite radiographic value the whole world over and which will greatly simplify our calculations. This unit we call the *milliampere-second*, and the beginner should get into the habit of "thinking in it" from the outset.

The amount of chemical action upon a photograpic plate is directly proportional to the quantity of rays that strike the plate. Obviously, therefore, an exposure of 60 milliampere-seconds will have the same effect upon a plate—whether it be made up of 6 milliamperes for 10 seconds, or 1 milliampere for 60 seconds, or 3 milliamperes for 20 seconds. At the end of this chapter a table will be given of exposures in milliampere-seconds suitable for various parts of the body. This table of "standard" exposures is based on average adult male parts, and it holds good only when a tube of suitable penetration is used at what we shall later define as the standard distance. Let us now see how the other factors may compel us to modify our standard exposure.

### The Distance Between Anticathode and Plate.

The simplest plan for the beginner is to work at first with the anticathode always at the same distance from the plate, and then this factor will not modify the standard exposure. A convenient

* Previous articles appeared on Nov. 11, 25, Dec. 9, 30, 1911, Jan. 13, 27, Feb. 17, March 9, 30, April 20, May 4, 25, June 8, July 6, Aug. 3, 17, 31, Sept. 28, Oct. 12, 26, and Nov. 9, 1912.

standard distance is *18 inches* (measured from the centre of the anticathode stem to the plane of the plate); a greater distance unnecessarily prolongs the exposure, a shorter distance produces undue distortion of the image.

Of course, when using a compressor cylinder, and in some cases where distortion is desired, it will be necessary to work with the anticathode nearer or farther from the plate than 18 inches. The rule then is that the exposure varies inversely as the *square* of the distance between anticathode and plate—hence, if we double our distance we must multiply our standard exposure (calculated for 18 inches) by four. It is a simple matter to square both distances and to convert the products into a vulgar fraction, by which the standard exposure must be multiplied, but to save this calculation the following figures may be useful. If we have decided that a certain exposure would be correct at a distance of 18 inches, then at 12 inches we should give one-half that exposure, at 15 inches two-thirds, at 21 inches we should multiply it by $1\frac{1}{3}$, at 24 inches multiply by $1\frac{2}{3}$, and so on.

### The Thickness of the Part.

This is the only factor that is not entirely under our control, and even this can be modified to a certain extent by compression. Since our exposure table is based upon parts of average dimensions it will, of course, be necessary to increase or diminish the standard exposure when we are confronted by a part that is stouter or thinner than the average, but with a little experience this becomes a simple matter.

Of course, when radiographing the limbs of young children, especially when it is desired to bring out developmental changes in bone and cartilage, it is necessary to use a softer tube as well as to reduce the exposure.

Trouble is, however, most likely to arise when one has to take the kidney or pelvis of a very obese individual; such cases tax to the utmost the skill of even experienced radiographers, and beginners should not be discouraged if they obtain poor results. The tube used should be a hard one, and it may be necessary even to double the standard exposure for a kidney. The difficulty is due not so much to the obstruction of the rays as to the generation of fresh *x*-rays (secondary rays) within the tissues themselves. It is in these cases that a compressor cylinder is particularly helpful.

### The Penetration of the Tube.

Throughout these articles *x*-ray penetration will be expressed in Wehnelt degrees. The Wehnelt radiometer was described on page 100, and there is good reason for adopting it as the universal standard of penetration since it is more uniformly graduated than any other type of instrument.

We shall consider penetration under two headings—its influence upon exposure, and its effect upon the quality of the radiograph.

*Its influence upon exposure* will not affect our standard figures provided we select a tube of suitable penetration, but if we are obliged to use a tube that is too soft we may to a certain extent compensate for this by increasing the exposure. This is possible because all tubes give out a small proportion of rays that are harder than their nominal penetration, and if we prolong the exposure we ensure that sufficient of these rays reach the plate to produce a satisfactory image. The practical outcome of this is that we may buy quite soft tubes, and, by using them for radiographing hands and toes and the limbs of small children, get a longer life out of them before they require to be regenerated.

It will be convenient to finish our consideration of exposure before discussing penetration in its other aspects.

### Table of Standard Exposures.

| | Milliampere-seconds |
|---|---|
| Fingers and metacarpals | 14 |
| Wrist and lower part of forearm | 20 |
| Elbow joint | 40 |
| Shoulder and upper end of humerus | 60 |
| Heart | 60 |
| Lungs | 40 |
| Head, lateral (to show orbit and accessory sinuses) | 60 |
| Head, postero-anterior | 140 |
| Teeth | 15 |
| Stomach and intestines (after a bismuth meal) | 120 |
| Kidney, to show calculi | 220 |
| Pelvis | 200 |
| Hip and upper end of femur | 120 |
| Knee joint | 60 |
| Ankle and tarsal bones (lateral view) | 40 |
| Toes and metatarsals | 20 |

These exposures are suitable for an average adult male subject, using a tube of proper penetration for the part (this will be indicated later), at a distance of 18 inches and with a diaphragm 2 inches in diameter.

It must be noted that the above figures represent *milliampere-seconds*—not seconds—consequently they must be divided by the number of milliamperes passing through the tube in order to arrive at the exposure in seconds. The normal current should be passed through the tube whenever possible, and if this current be two milliamperes the correct exposure in seconds will be one-half the above numbers; if five milliamperes are passing through the tube the exposure in seconds will be one-fifth of the above, and so on until, if our apparatus be powerful enough and we have a tube that will stand so strong a current, we can reduce the exposure to a small fraction of a second for most parts of the body and still give the full exposure in milliampere-seconds (*e.g.* 200 milliamperes × $\frac{1}{5}$ second = 40 milliampere-seconds). This, however, takes us into the realms of instantaneous radiography with which we are not at present concerned.

The above exposure table gives results with which the writer is satisfied, but every beginner should compile a table for himself as he goes along. He can best do this by keeping an exposure register and noting down the details of every exposure he makes; it is then an easy matter to select his most perfect results and tabulate them for future guidance. The register should be printed in columns under the following headings: Date; No.; Name; Age; Part; Thickness; Direction; Tube; Penetration; Distance; Milliampere-seconds.

(*To be continued.*)

